# Comparative expression analysis of aquaporin-5 (AQP5) in keratoconic and healthy corneas

**Published:** 2008-04-25

**Authors:** Yonathan Garfias, Alejandro Navas, Hector J. Pérez-Cano, Jonathan Quevedo, Leonardo Villalvazo, Juan Carlos Zenteno

**Affiliations:** 1 Research Unit, Institute of Ophthalmology “Conde de Valenciana,” Mexico City, Mexico; 2 Department of Cornea, Institute of Ophthalmology “Conde de Valenciana,” Mexico City, Mexico; 3 Department of Pathology, Institute of Ophthalmology “Conde de Valenciana,” Mexico City, Mexico; 4 Department of Genetics, Institute of Ophthalmology “Conde de Valenciana,” Mexico City, Mexico

## Abstract

**Purpose:**

Keratoconus (KC) is a common progressive corneal disease characterized by excessive stromal thinning, central or paracentral conical protrusion, and disruptions in Bowman’s layer. The etiology of KC is largely unknown, and a combination of genetic and environmental factors is believed to play a role in the origin of the disease. Recently, the absence of transcripts of the water channel, aquaporin-5 (AQP5), was demonstrated by reverse-transcription polymerase chain reaction (RT–PCR) in KC tissues and was proposed as a possible marker for KC. In this study, we sought to evaluate *AQP5* mRNA and protein expression in KC and non-KC corneal tissues using a combination of techniques.

**Methods:**

A total of 69 samples of corneal tissue were analyzed including 39 corneal buttons from patients with advanced KC, 16 samples of non-KC corneal epithelium belonging to patients who underwent surface refractive surgery, 12 sclerocorneal rims obtained from healthy donor subjects, and two healthy corneal buttons. Determination of *AQP5* transcript and protein expression patterns was performed by means of real time RT–PCR, immunohistochemistry, immunocytochemistry, and flow cytometry methods. Cell culture was performed to identify AQP5 protein expression in KC epithelial cells.

**Results:**

*AQP5* mRNA was expressed with no significant differences between KC and non-KC tissues. Moreover, AQP5 protein expression analysis did not reveal differences in protein levels and/or cell location among KC and non-KC tissues. Interestingly, AQP5 expression continues for up to 21 days in the isolated KC corneal epithelial cells.

**Conclusions:**

Our results do not support a role for AQP5 in KC etiopathogeny or as a disease marker. Genetic background differences or a distinct pathogenetic KC cascade specific to the analyzed population could account for the dissimilarities observed in KC-related *AQP5* expression.

## Introduction

Keratoconus (KC) is a heterogeneous disorder in which the cornea assumes a conical shape as a consequence of a gradually progressive non-inflammatory thinning of the corneal stroma. Corneal thinning in KC individuals induces irregular astigmatism, myopia, and central or paracentral conical protrusion. The calculated incidence of KC is between 1 in 500 and 1 in 2,000 individuals in the general population [1] with the disease being the most common indication for penetrating keratoplasty in developed countries [2]. Histopathological features of keratoconic corneas have been clearly defined and include stromal thinning, iron deposits in the epithelial basement membrane, and breaks in Bowman's layer [3]. The etiology of KC is largely unknown, and genetic and environmental factors are believed to play a role in distinct analyzed populations [4]. Most cases of KC occur sporadically, but it has been estimated that up to 10% of cases has a positive familial history for the disease [5]. This observation along with the occurrence of the disorder in monozygotic twins and the bilateralism of the disease strongly suggest that genetic factors are important for KC development. In families with KC Mendelian transmission, autosomal dominant and autosomal recessive inheritance has been established. In addition, genome-wide linkage analyses in familial cases have evidenced several chromosomal regions that harbor potential KC-causing genes including 5q14.3-q21.1, 16q22.3-q23.1 [6], 3p14-q13 [7], and 2p24 [8]. However, a definitive KC-causing gene has not been identified to date. As the disease initiates typically during adolescence, the identification of a molecular marker associated to the development of the disease would be particularly helpful in the early identification of individuals at risk of developing KC.

The aquaporins (AQPs) constitute a large family of water channels that play a critical role in transcellular water movement in many tissues [9]. The aquaporin-5 (*AQP5*) gene is normally expressed in corneal epithelial cells where it is presumably involved in fluid elimination as supported by the observation that *Aqp5* null mice exhibit abnormalities in both corneal thickness and corneal epithelial water permeability [10]. Recently, the absent expression of *AQP5* from the corneal epithelium was shown to be a feature of KC corneas [11]. However, no additional studies have been performed to validate the possible involvement of *AQP5* in KC. To further investigate this issue, we analyzed the expression of this gene in KC and healthy corneal tissues using immunohistochemistry, real time polymerase chain reaction (PCR), flow cytometry, and immunocytochemistry.

## Methods

### Corneal samples

Sixty-nine samples of corneal tissue were obtained; 39 samples were corneal buttons from patients with KC, 16 were samples of corneal epithelium of patients who underwent surface refractive surgery (Photorefractive Keratectomy or Epi-LASIK), 12 were sclerocorneal rims obtained from healthy donor subjects, and two buttons were from healthy corneas. In the keratoconus group, the mean age was 25 years (range 14–48 years), 68% were male and 32% were female. All KC cases correspond to advanced or late-stage instances of the disease confirmed by clinical (central or paracentral leukomas, Vogt’s striae, and severe corneal stromal thinning) and corneal topographic criteria. Patients gave the appropriate permissions for their corneas to be used for this research. All tissues were obtained within 24 h after surgery. Samples were rinsed with 0.15 M phosphate-buffered saline (PBS) and divided into two pieces unless otherwise stated. One fragment of each sample was paraffin embedded whereas the other fragment was used for molecular analysis.

**Figure 1:**
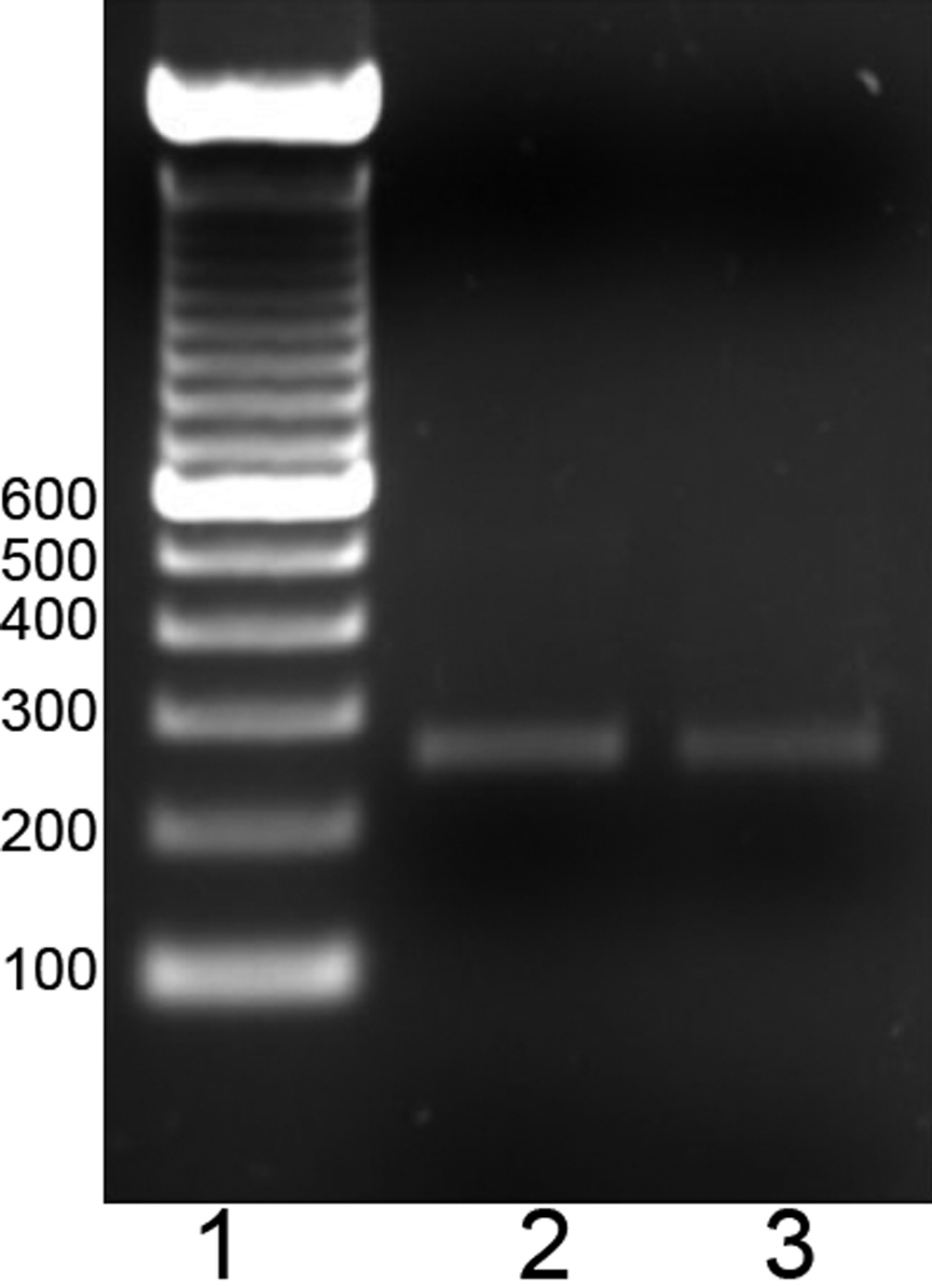
Electrophoretic analysis of conventional AQP5 reverse-transcription polymerase chain reaction in keratoconus and non-keratoconus corneal tissue. Amplicons for *AQP5* were visualized in a 1.5% agarose ethidium bromide gel from both samples KC and non-KC corneal tissue. Lane 2, corresponds to *AQP5* amplicon from KC corneal tissue; lane 3, corresponds to *AQP5* amplicon from non- KC corneal tissue. Molecular weight markers are shown in lane 1. Similar bands intensities are observed.

### Tissue RNA extraction and conventional reverse-transcription polymerase chain reaction

RNA extraction from 10 KC and 7 non-KC corneal samples was achieved using the RNAeasy kit (Qiagen, Heiden, Germany) as indicated by the manufacturer. Retrotranscription was performed with the Omniscript kit (Qiagen) according to the manufacturer’s instructions. The RNA concentration was evaluated by A260/A280 measurement and was retrotranscribed at 37 °C for 1 h in the presence of oligo dT (Invitrogen, Carlsbad, CA). The PCR reaction for *AQP5* was performed with Hotstart DNA Polymerase (Qiagen) using the following pair of primers, forward 5′-CGT TTG GCC TGG CCA TAG GCA−3′ and reverse 5′-TGG CCC TGC GTT GTG TTG TTG−3′. Band intensities of PCR products were quantified by densitometric scanning using the Kodak 1D Image Analysis software (Kodak, Rochester, NY), and values were normalized against amplification of *GAPDH* as a constitutive gene.

### Real time polymerase chain reaction

From the obtained cDNA, real time PCR was performed using the phosphoglycerate kinase 1 (*PGK-1*) gene as previously described [12]. Up to 5 ng of starting RNA was used for amplification in a Rotor-Gene 6000 apparatus (Corbett Life Science, Sidney, Australia). Primers for *PGK-1* and *AQP5* amplification had the same melting temperature and were *PGK-1* forward 5′-ATT AGC CGA GCC AGC CAA AAT AG-3′, *PGK-1* reverse 5′-TCA TCA AAA ACC CAC CAG CCT TCT-3′, *AQP5* forward 5′-CAT CTT CGC CTC CAC TGA CT-3′, and *AQP5* reverse 5′-CCC TAC CCA GAA AAC CCA GT-3′. To identify that the amplification reactions did not form any spurious sub-products, the temperature gradient was performed from 75 °C to 95 °C and was analyzed by melt curve. In all cases, only one curve was obtained for each amplification reaction. Relative amplification increment was calculated using the formula, AQP5Ct/PGK1Ct. Each experiment was done in triplicate in five independent assays. Comparisons between groups were performed using the Student’s unpaired *t*-test and were considered to be significant if p<0.05.

**Figure 2:**
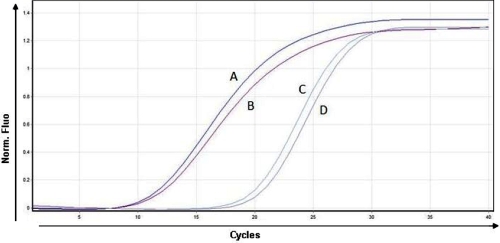
*AQP5* real time polymerase chain reaction comparative analysis in keratoconus and non-keratoconus corneal tissue. PCR products from both samples KC and non-KC corneal tissue are shown. Curves A and B correspond to the *PGK-1* gene amplification from healthy and KC corneal tissue, respectively. Curves C and D correspond to *AQP5* amplification from healthy and KC corneal tissue, respectively. The x-axis represents the number of cycles, and the y-axis represents normalized fluorescence. The graph is a representative example of five separate assays.

### Automated DNA sequencing

*AQP5* products amplified by reverse-transcription (RT)–PCR were directly sequenced to confirm the identity of the amplicon. Nucleotide sequence was achieved using the BigDye terminator kit (Applied Biosystems, Foster City, CA) of fluorescently labeled terminators and analyzing the samples in an ABI 310 DNA Sequencer (Applied Biosystems). Sequences were compared to the published human wild type *AQP5* gene (Ensembl transcript ID number ENST00000293599).

### Immunohistochemistry

Twenty-nine KC and 14 non-KC specimens were analyzed by immunohistochemistry. Histological sections (5 μm thick) that were collected on poly-L-lysine-coated slides were deparaffinized; the sections were rehydrated in a xylene-graded alcohol scale and then rinsed with PBS. Intrinsic peroxidase activity was blocked with 3% H_2_O_2_ for 5 min at room temperature and then exposed to primary goat anti-human AQP5 antibody (Santa Cruz Biotechnology, Santa Cruz, CA) for 1 h at room temperature. After washing in 0.05% Tween 20-PBS, the sections were exposed to universal biotin conjugated antibodies for 30 min at room temperature. The sections were incubated with peroxidase-conjugated streptavidin for 20 min at room temperature. The color development reaction was performed with diaminobenzidine chromogen and 0.05% H_2_O_2_. The sections were counterstained with Meyer’s hematoxylin (Dako, Glostrup, Denmark). Human tonsil tissue for AQP5 immunohistochemistry were used as a positive control as previously described [13]; negative controls were tissue samples in which the primary antibody (AQP5) was not added.

**Figure 3:**
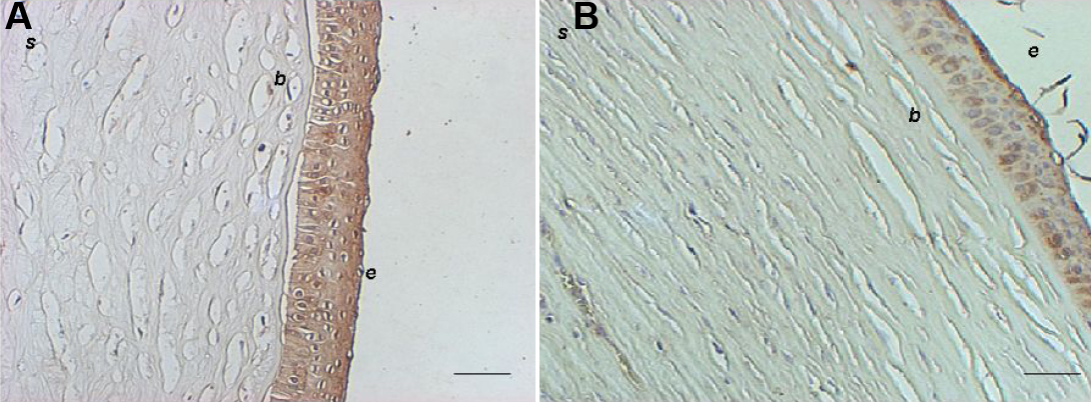
AQP5 immunohistochemistry from keratoconus and non-keratoconus corneal tissue. **A**: The immunopositive reaction to AQP5 from a KC sample is shown as a brown signal in the epithelial cells. **B**: Non-KC tissue demonstrates a similar immunopositive AQP5 reaction. Note disruptions in Bowman’s layer in **A**. b, Bowman’s layer; e, corneal epithelium; s, stroma (Representative assay of 29 KC and 14 non-KC samples). Bar=100 µm.

### Cell culture and immunocytochemistry

Corneal tissue obtained from surgery was rinsed with PBS twice and incubated with 1.2 IU/ml of Dispase II (Roche, Mannheim, Germany) for 30 min at 37 °C. Five KC and five non-KC tissue samples were cut into four pieces and seeded in 24 well plastic culture plates for 20 min at 37 °C. A drop of 25 μl of heat inactivated bovine fetal serum (Gibco, Carlsbad, CA) was subsequently added to the tissue. After 24 h of incubation, 1 ml of keratinocyte serum free medium (Gibco) was added and changed every other day. Cell culture was allowed until 90% of cell confluence was reached. As the cell culture control, limbal epithelial cells were cultured as described by Luna-Baca et al. [14]. Cultured cells were attached into 12 mm diameter cover glasses for 24 h at 37 °C in 5% CO_2_. Afterwards, the cover glasses were fixed by adding ice-cold absolute ethanol for 10 min. Intrinsic peroxidase activity was blocked with 3% H_2_O_2_ for 5 min at room temperature and then exposed to primary goat ant-human AQP5 antibody for 1 h at room temperature. After washing in 0.05% Tween-PBS, the sections were exposed to universal biotin-conjugated antibodies for 30 min at room temperature. The sections were incubated with peroxidase-conjugated streptavidin for 20 min at room temperature. The color development reaction was performed with diaminobenzidine chromogen and 0.05% H_2_O_2_. The immunoreaction was assessed under light microscope.

**Figure 4:**
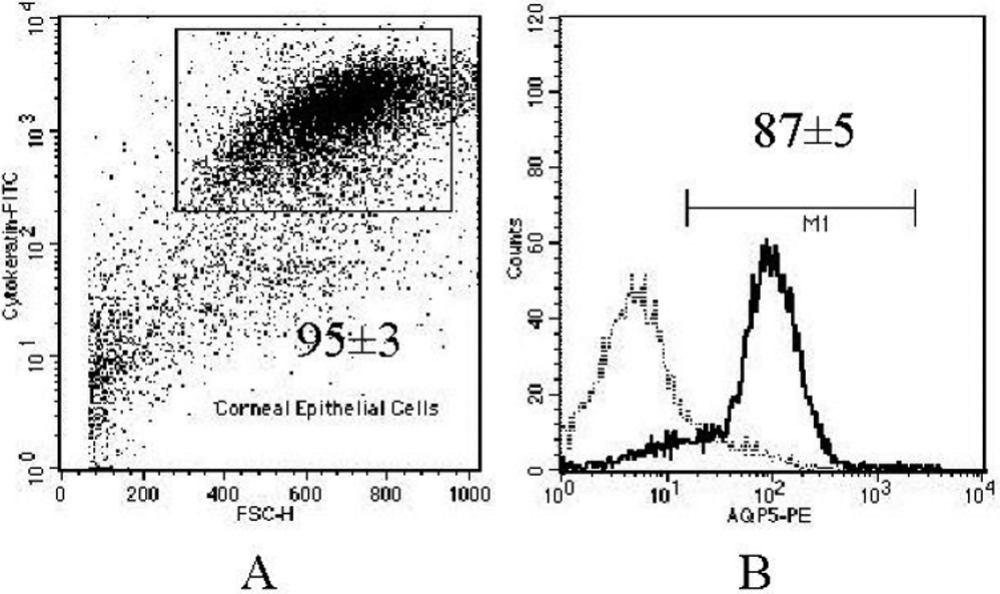
AQP5 flow cytometry in epithelial cells from keratoconus cornea. **A**: Flow cytometry dot plot from single cell suspension from KC cornea labeled with anti-cytokeratin. The inner rectangle represents cytokeratin positive cells **B**: AQP5 flow cytometry histogram is shown from cytokeratin positive cells. The continuous dark line represents AQP5+ cells; the dashed light line represents the negative control. Inner numbers indicate media ± SEM (n=5).

### Flow cytometry

Five KC and five non-KC corneal tissues were incubated with 1.2 IU/ml Dispase II for 45 min at 37 °C. After the addition of PBS, the cell suspension was extensively triturated through a fire-polished Pasteur pipette and sequentially filtered through 40 μm and 10 μm nylon mesh sieves. Five-hundred microliters of 4% p-formaldehyde (Sigma Aldrich, St Louis, MO) were added and incubated for 10 min at 4 °C. Cells were washed and incubated with 10 μl of anti-human AQP5 (Santa Cruz Biotechnology) and anti-human cytokeratin (Dako) antibodies for 30 min at room temperature. After washing the cells twice, the pellet was incubated with 20 μl of secondary fluorochrome-conjugated antibodies (BD PharMingen, San Jose CA). The cells were then analyzed by flow cytometry in a FACScalibur apparatus (Becton Dickinson, La Jolla, CA). Suitable negative controls were used.

### Western blot

Total protein was obtained from corneal KC and non-KC epithelial cells following standard conditions (details on request). Proteins were separated by electrophoresis on 12% SDS-polyacrylamide gel and were transferred to a nitrocellulose membrane. The membrane was pre-incubated with blocking buffer (5% non-fat milk, 0.05% Tween 20, and TBS (100 mM Tris HCl, pH 8.0, 150 mM NaCl) for 2 h at room temperature and incubated with polyclonal goat anti-AQP5 antibody at 4 °C overnight. The membrane was then washed in several changes of washing buffer (0.05% Tween 20 in TBS), incubated for 2 h with mouse anti-goat biotin-conjugated immunoglobulins at room temperature. The membrane was washed in several changes of washing buffer and incubated with anti-biotin, peroxidase-conjugated Fab fragments for 1 h at room temperature. Proteins were detected using the Western Lightning Chemiluminescence reagent (Perkin Elmer Life Science, Boston, MA).

**Figure 5:**
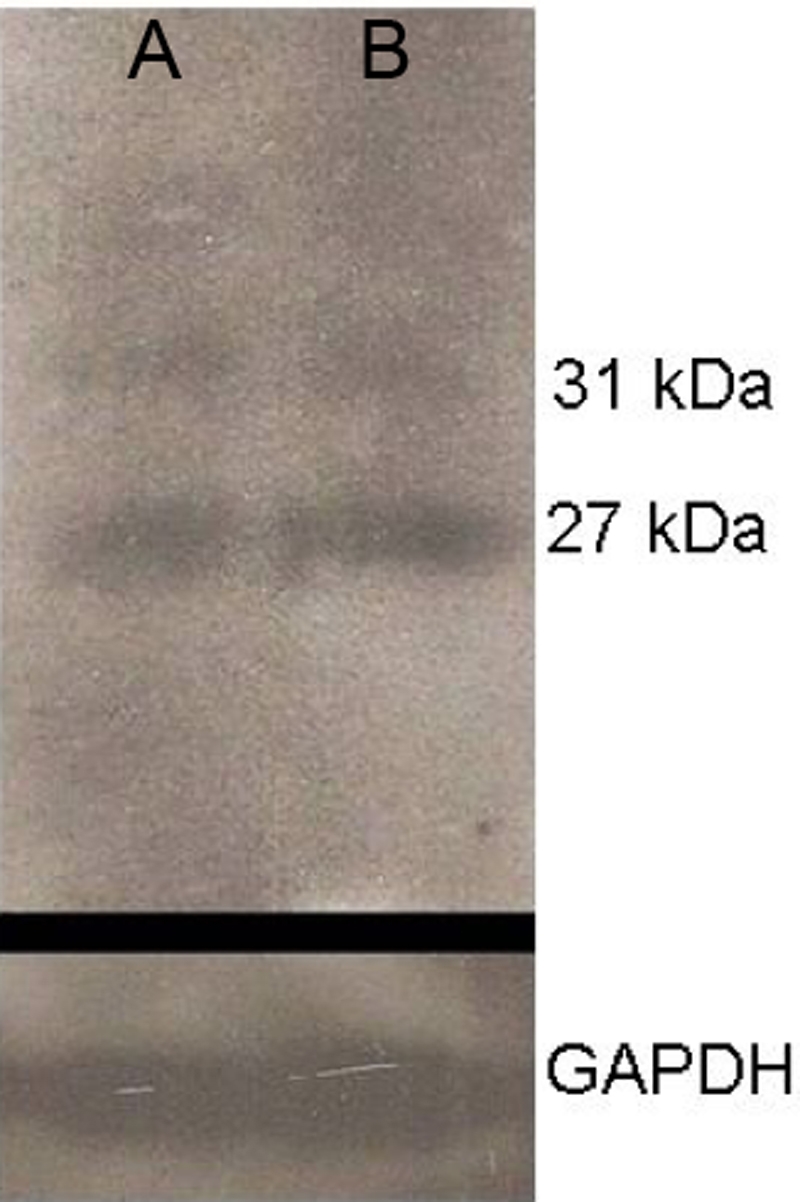
Western blot for AQP5 in keratoconus and non-keratoconus epithelial cells. The photograph of a western blot identifies two bands of AQP5. Twenty-seven and 31 kDa corresponds to the non-glycosylated protein and glycosylated form of the AQP5 protein, respectively. Lane A corresponds to a non-KC sample; lane B corresponds to a KC sample. GAPDH protein is shown below as an internal control.

## Results

### Determination of *AQP5* transcripts in corneal epithelial cells

Band intensities of PCR products were compared by densitometric analysis (Figure 1). No statistically significant differences (p>0.05) were observed between KC (n=10) and non-KC corneal tissue (n=14). To determine if differences in the amplification reaction due to distinct initial RNA concentration existed, real time PCR was performed, normalizing with a constitutive *PGK-1* gene. The average relative increment of samples from both groups ranged between 1.01 and 1.05 with no statistically significant differences (p>0.05; Figure 2).

### Nucleotidic sequence

The nucleotide sequence of the *AQP5* amplicon disclosed a 100% homology with the wild type *AQP5* sequence.

**Figure 6:**
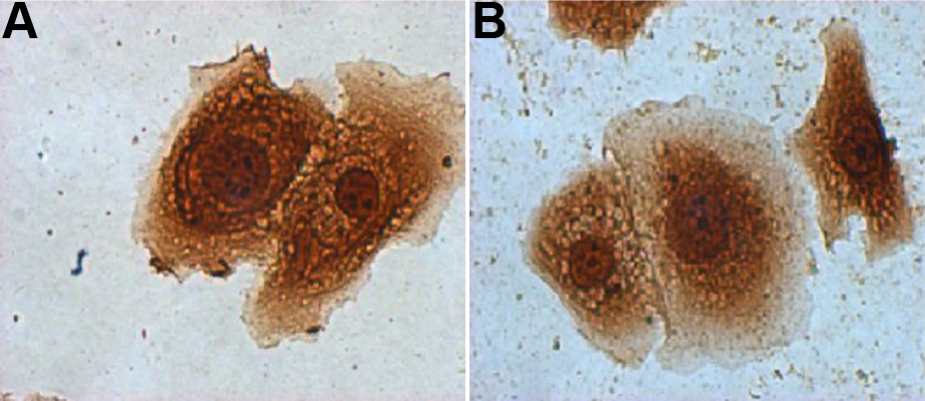
AQP5 immunocytochemistry of cultured corneal epithelial cells. **A**: Epithelial cells obtained from a cell culture of KC corneas strongly immunoreact to AQP5. **B**: Epithelial cells cultured from non-KC tissue display a similar pattern of AQP5 immunostaining. This figure is representative of five separate assays.

### Corneal epithelial cells from keratoconus ex vivo and in vitro express constitutively AQP5 protein

Immunohistochemistry was performed on 29 samples of KC corneal epithelium, 12 samples of sclerocorneal rims, and two healthy corneas. Under light microscopy, no apparent differences in the pattern of immunoreaction were observed comparing KC cells with normal corneal epithelial cells (Figure 3). When the cell suspension of epithelial cells from KC was analyzed by flow cytometry, almost 95% of cells were cytokeratin positive, indicating that the major population of cells obtained from corneal tissue treated were epithelial cells. From those epithelial cells, approximately 90% of the cells were positive to AQP5 (Figure 4A,B). Western blot analysis revealed the identification of two bands of similar intensity in both KC and non KC samples (27 and 31 kDa, corresponding to the non-glycosylated and glycosylated forms of the AQP5 protein, respectively; Figure 5). Similarly, when immunocytochemistry assays were performed in corneal epithelial cells from KC and non-KC tissue grown in vitro, all analyzed cells expressed constitutively the AQP5 protein. A similar pattern of AQP5 immunostaining was observed in these KC and non-KC cells (Figure 6).

## Discussion

Aquaporin water channels are expressed primarily in cell plasma membranes where they function as selective pores allowing water, glycerol, and other small solutes to pass through the cell membrane. AQP5 plays an important role in corneal fluid elimination via the corneal epithelium [15], and *Aqp5* null mice have increased corneal thickness due to corneal swelling [10]. These data along with the finding of the absence of *AQP5* transcripts from KC corneas [11] have led to a proposal that there is a potential role for AQP5 in the physiopathology of KC. In this study, we sought to compare the expression of AQP5 in KC and non-KC corneal tissues by means of several different techniques. Our results indicate that *AQP5* expression levels are similar in KC and non-KC corneas and that the immunolocalization of AQP5 did not differ between both groups when analyzed by immunohistochemistry and immunocytochemistry. In our study, corneal epithelial tissues from non-KC subjects were compared to corneal buttons from KC. The difference in tissue samples (corneal epithelium versus corneal buttons) did not affect our conclusions as it has been demonstrated that AQP5 is exclusively expressed in corneal epithelial cells [15].

Controversial results have been reported regarding the involvement of AQP5 in KC. Kenney et al. [16] found no significant differences in the staining pattern for AQP5 in epithelial cells of normal corneas and KC corneas. In contrast, another study reported the absence of *AQP5* transcripts from KC tissues when analyzing clones from a human KC cornea cDNA collection and by RT–PCR [11]. This finding was interpreted as a possible feature of KC corneas. However, the comprehensive evaluation of both *AQP5* mRNA and protein in KC tissues that we describe in this work argue against a potential involvement of this aquaporin as a KC etiologic factor or as a late-stage marker of the disease. The discrepancy between our results and those previously published could be explained by specific genetic differences between ethnic groups, by the stage of KC development analyzed, which was moderate to advanced KC in the Rabinowitz et al. study [11] and exclusively advanced KC in our study, or by distinct etiopathologic factors among KC subjects from different populations.

Altogether, these data indicate that an abnormality in AQP5 mRNA or protein is not likely involved in KC physiopathology. In addition, to determine the possibility of *AQP5* mutations occurring in DNA from KC patients, a nucleotidic sequence of the *AQP5* gene was performed in several individuals with the disease, but no mutations were identified [unpublished data]. This result is in agreement with those reported by Rabinowitz et al. [11] who did not find *AQP5* mutations in DNA from KC corneas.

In our study, AQP5 staining by immunohistochemistry revealed a punctate rather than a membrane-associated epithelial pattern. This pattern is identical to that reported previously [16]. An interesting find in this work was that AQP5 expression is preserved in KC corneal epithelial cells after 21 days of culture. This result suggests AQP5 could be used as a suitable marker for cultured corneal epithelial cells. In conclusion, we do not find evidence of AQP5 involvement in KC corneal tissue in this sample of Mexican patients.
